# Beneficial effects of Red Light-Emitting Diode treatment in experimental model of acute lung injury induced by sepsis

**DOI:** 10.1038/s41598-017-13117-5

**Published:** 2017-10-04

**Authors:** Silvia Goes Costa, Éric Diego Barioni, Aline Ignácio, Juliana Albuquerque, Niels Olsen Saraiva Câmara, Christiane Pavani, Luana Beatriz Vitoretti, Amílcar Sabino Damazo, Sandra Helena Poliselli Farsky, Adriana Lino-dos-Santos-Franco

**Affiliations:** 1 Post Graduate Program in Biophotonics Applied to Health Sciences, University Nove de Julho (UNINOVE), São Paulo, Brazil; 2 Department of Clinical and Toxicological Analyses, Faculty of Pharmaceutical Sciences, University of São Paulo, São Paulo, Brazil; 3 Department of Immunology, Institute of Biomedical Sciences, University of São Paulo, São Paulo, Brazil; 4 Department of Basic Science in Health, Faculty of Medical Sciences, Federal University of Cuiabá, Cuiabá, Brazil

## Abstract

Sepsis is a severe disease with a high mortality index and it is responsible for the development of acute lung injury (ALI). We evaluated the effects of light-emitting diode (LED) on ALI induced by sepsis. Balb-c mice were injected with lipopolysaccharide or saline and then irradiated or not with red LED on their tracheas and lungs for 150 s, 2 and 6 h after LPS injections. The parameters were investigated 24 h after the LPS injections. Red LED treatment reduced neutrophil influx and the levels of interleukins 1β, 17 A and, tumor necrosis factor-α; in addition to enhanced levels of interferon γ in the bronchoalveolar fluid. Moreover, red LED treatment enhanced the RNAm levels of IL-10 and IFN-γ. It also partially reduced the elevated oxidative burst and enhanced apoptosis, but it did not alter the translocation of nuclear factor κB, the expression of toll-like receptor 4 (TLR4), as well as, oedema or mucus production in their lung tissues. Together, our data has shown the beneficial effects of short treatment with LED on ALI that are caused by gram negative bacterial infections. It is suggested that LED applications are an inexpensive and non-invasive additional treatment for sepsis.

## Introduction

Acute lung injury (ALI) is a severe and serious disease. It is multifactorial and characterized by diffused alveolar damage, lung inflammatory cell infiltrations, as well as a loss of alveolar epithelium, together with oedema and an impaired gas exchange^1^. ALI is associated with a high mortality incidence around the world (200,000 patients per year in the US alone, resulting in a mortality rate of 40%)^1^.

ALI is divided in exudative and fibro proliferative phases. The first one is characterized by an activation of the coagulation system, with a production of pro-inflammatory cytokines by the epithelial tissues, together with the resident mast cells, the macrophages, and the recruitment of neutrophils, monocytes and macrophages, as well as lymphocytes, into the lung injury. Furthermore, the release of cytotoxic mediators, reactive oxygen species (ROS), reactive nitrogen species (RNS), proteolytic enzymes, as well as metalloproteinases (MMPs), cause an endothelial and epithelial lung injury, impacting in the loss of functions of lung^2,3^. The fibro proliferative phase occurs approximately 3 days after the injury, with a predominant proliferation of mesenchymal cells, such as myofibroblasts, fibroblasts, and pluripotent cells.

Sepsis is an important clinical condition that is considered to be the most important cause of ALI developments. Sepsis is a systemic response to infection that is manifested by changes in the body temperature and tachycardia, together with elevations in the respiratory frequency, leukocytosis in the lungs, and blood leukopenia^4^. ALI has been experimentally induced by systemic administration of lipopolysaccharide (LPS), which interacts with toll-like receptors (TLR), mainly of type 4 (TLR_4_)^5^. The activation of TLR_4_ downstream leads to complex signal transduction pathways that induce the translocation of nuclear factor κ B (NF-κB) into the nuclei. NF-κB is responsible for the transcription of the pivotal inflammatory genes^6^. Indeed, a pharmacological inhibition of the NF-κB nuclear translocation leads to an impairment of the inflammatory responses to LPS^7^.

Neutrophils are target cells in ALI that are evoked by sepsis, as circulating cells are recruited into the lung in the early phases of the syndrome^8^. As a consequence, the activated neutrophils secrete cytokines and chemokines, releasing granule contents as proteases, producing reactive oxygen (ROS) and nitrogen (RNS) species, which contribute to the amplification of the inflammation and tissue damage^9^. When considering the crucial role of neutrophils in an ALI, some studies have proposed to induce neutrophil apoptosis as a strategy of treatment^10,11^.

The treatment of ALI is a clinical problem, as anti-inflammatory drugs are inefficient^3,12–14^. Additional approaches have been integrated into the therapy, such as mechanical ventilations with a low volume, a prone position (face down), as well as an extracorporeal membrane oxygenation (ECMO). Nevertheless, these kinds of treatments all require high costs and they are not sufficiently effective^3,12–14^. As a result, treatments that are more efficient and therapies with lower costs are required in order to treat ALI.

Photobiomodulation is a treatment that is based on the effects of light on damage tissues, such as lasers, light-emitting diode (LED), among others. Photobiomodulation has been pointed out as an interesting tool for the treatment of lung diseases, as experimental studies have shown that a low level of laser therapy reduces the inflammation and oxidative stress in lung disorders^15–20^. However, the beneficial effect of LED in the lung diseases, such as experimental model of asthma^21^ and lung fibrosis^22^ has been shown by our group. The red LED treatment in asthmatic mice reduced the lung cell infiltration, mucus production, oedema, and tracheal’s contractile response by IL-10, IFN-у and mast cells mechanisms-involved^21^. We also showed that the red LED treatment reduced the number of inflammatory cells in the alveolar space, collagen production, interstitial thickening, and static and dynamic pulmonary elastance in experimental model of lung fibrosis. In addition, reduced levels of IL-6 and CXCL1/KC released by cultured pneumocytes as well as decreased secretion of CXCL1/KC by fibroblasts in culture^22^.

Moreover, recent data has shown that LED treatment have inhibited the release of inflammatory mediators in experimental model of arthritis. They have abrogated the mechanical and thermal hyperalgesia that was modulated by tumor necrosis factor-α (TNF-α), interleukins IL1-β (IL1-β) and 10 (IL-10), in murine experimental models with chronic inflammatory hyperalgesia. They have also inhibited cytokine secretions in human fibroblasts^23–25^.

Therefore, based on the beneficial effects of LED treatment in inflammatory diseases and in our previous studies, we have hypothesized that the LED treatment could act as anti-inflammatory agent in the lungs and be employed as an additional treatment for sepsis. Using an experimental model of sepsis that was induced by LPS in mice, we have investigated the effects of LED treatment on the initial course of ALI. The research has focused on leukocyte influxes into the lung, taking note of the local gene expression, the secretion of cytokines, oedema, mucus production, NF-κB nuclear translocation, oxidative burst, phagocytosis, as well as the gene expression of TLR4.

## Results

### Local repercussions of LED treatment in experimental model of ALI

**Figure 1:**
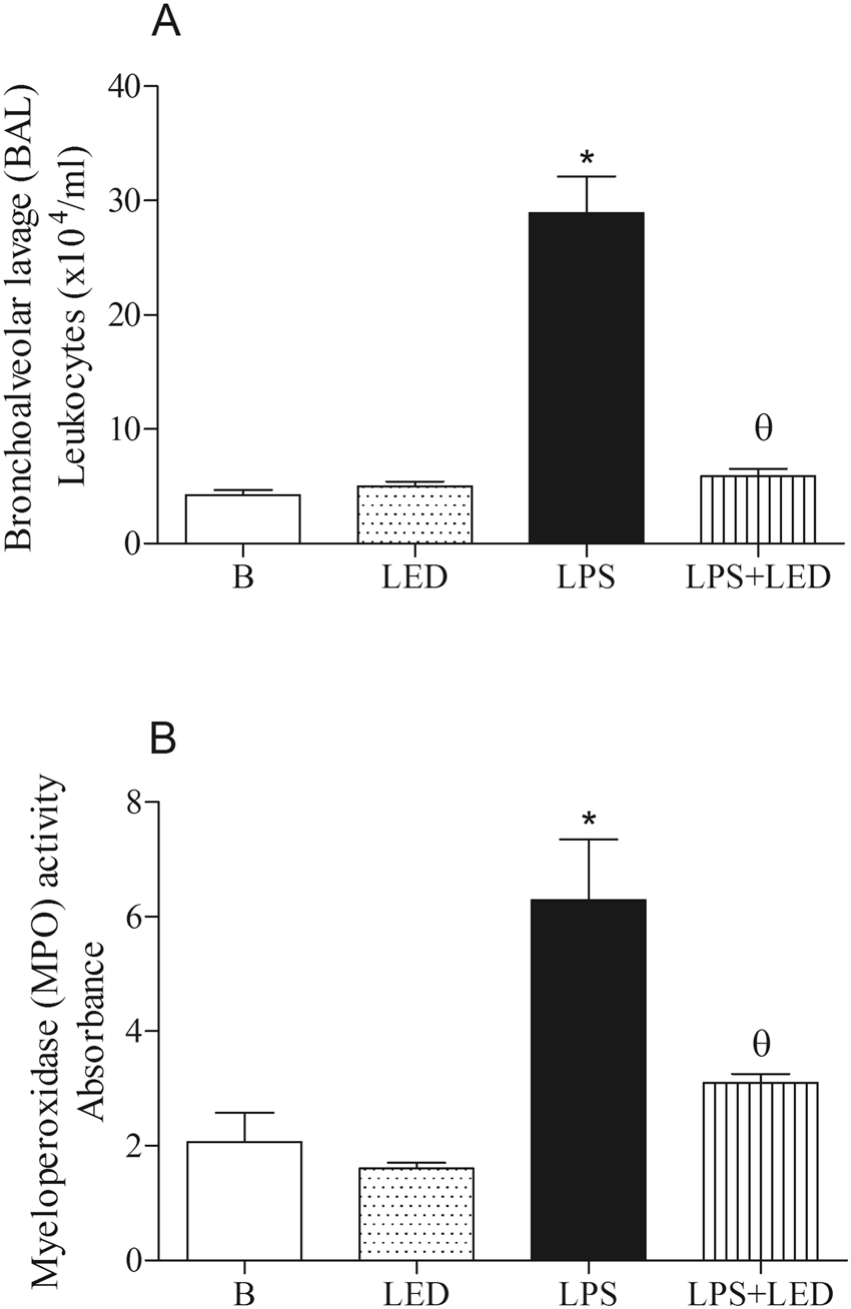
LED treatment decreases lung cell recruitment in the bronchoalveolar lage (BAL) and myeloperoxidase (MPO) activity in the lung tissue in ALI experimental model. Groups of mice were induced to ALI by ip injection of LPS and treated or not with LED 2 and 6 h after the induction. Non-manipulated and LED treated mice were used as control. After 24 hours of ALI induction, the cellular recruitment and the MPO activity were determined. Data mean ± SEM of 6 animals per group. *P < 0.05 in relation to B group; ^θ^P < 0.05 in relation to LPS group.

Data presented in Fig. 1 (Panel A) show that i.p. injection of LPS caused lung inflammation, characterized by elevated number of leukocytes, which include macrophages, lymphocytes and neutrophils in the BAL in comparison to number of cells found in non-manipulated mice (Basal). The treatment with LED in LPS injected mice reduced the number of leukocytes in the BAL, and numbers were rescued to those equivalent in Basal group (Fig. 1A). Moreover, LED treatment did not modify the number of cells in the BAL in absence of inflammation (Fig. 1A).

Furthermore, LPS injection enhanced the myeloperoxidase (MPO) activity the BAL, which is an index of accumulation of activated neutrophils in the BAL during 24 hours of sepsis^26,27^. Activity of MPO was higher in LPS group of animals when compared to Basal or LED treated groups. Corroborating the inhibitory effect of LED on neutrophil recruitment, lower MPO activity was detected in LPS injected and LED treated animals (Fig. 1).

### Systemic repercussions of LED treatment in experimental model of ALI

**Figure 2:**
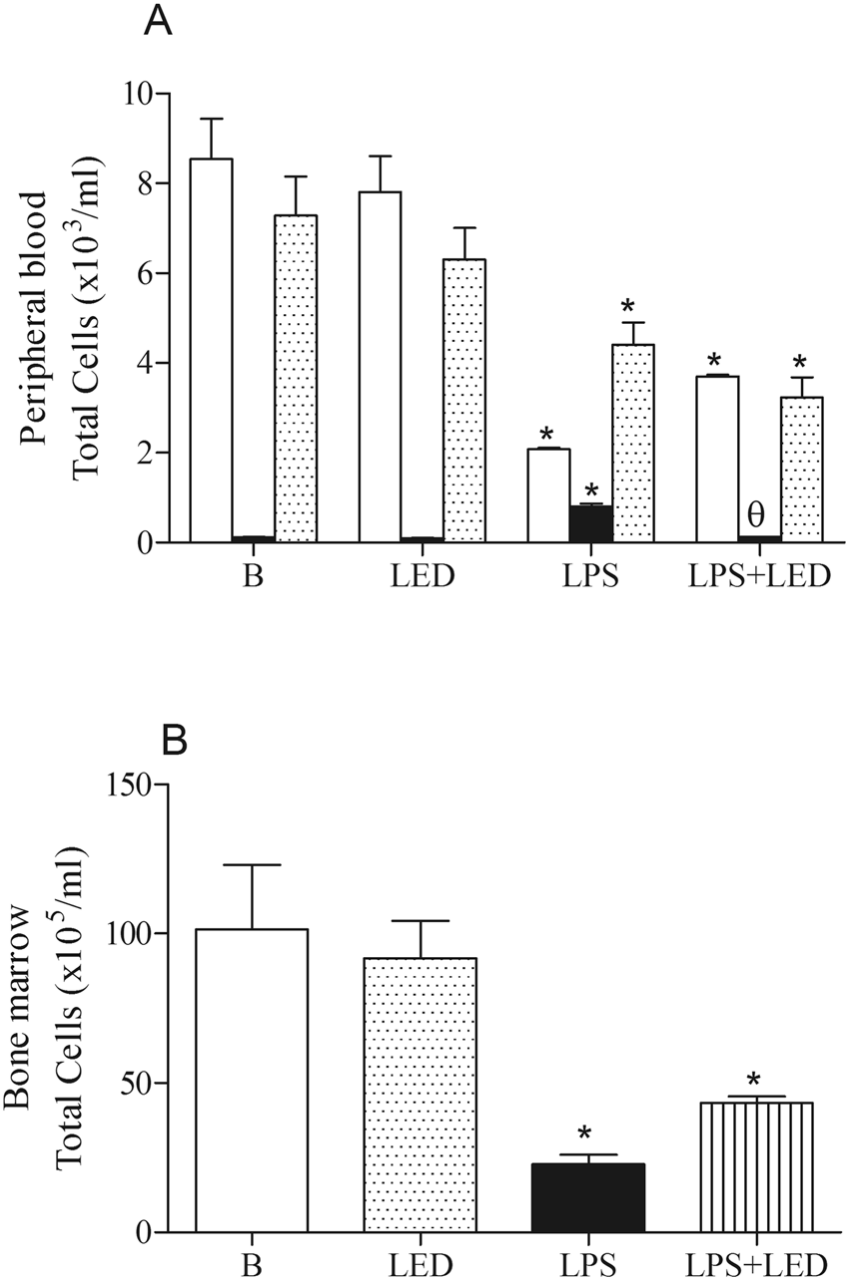
LED treatment decreases granulocytes in the blood without alter the number of cells in the bone marrow in ALI experimental model. Groups of mice were induced to ALI by ip injection of LPS and treated or not with LED 2 and 6 h after induction. Non-manipulated and LED treated mice were used as control. After 24 hours of ALI induction, the number of cells in the blood and in the bone marrow were quantified. Data mean ± SEM of 6 animals per group. *P < 0.05 in relation to B group; ^θ^P < 0.05 in relation to LPS group.

Further systemic analysis also characterized the LPS injection as a sepsis model, as LPS administration caused leukopenia, with drastic drop of mononuclear cells and enhanced number of granulocytes in the blood when compared to control groups (non-manipulated or LED treated mice). LED treatment did not modify the leukopenia and reduced number of mononuclear cells, but decreased the enhanced number of granulocytes caused by LPS injection (Fig. 2A).

Data presented in Fig. 2B showed that LPS administration reduced the number of leukocytes in the bone marrow when compared to the Control groups (Basal or LED treated mice) and, the treatment with LED did not rescue the reduced amount of cells in the bone marrow.

### The role of LED treatment in the cytokines production in experimental model of ALI

**Figure 3:**
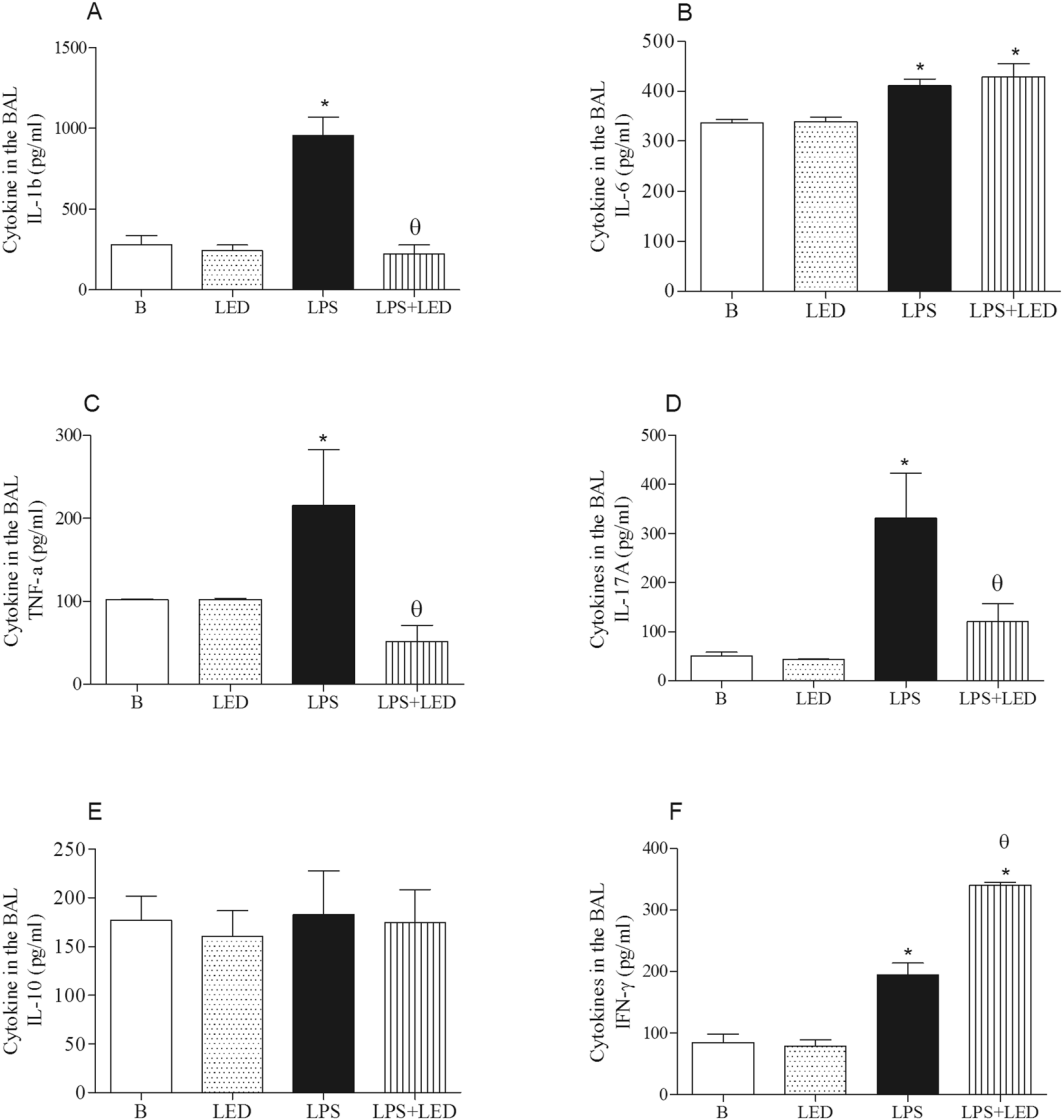
LED treatment increases the levels of anti-inflammatory cytokines and decreases the levels of pro-inflammatory cytokines in the BAL fluid in ALI experimental model. Groups of mice were induced to ALI by ip injection of LPS and treated or not with LED 2 and 6 h after induction. Non-manipulated and LED treated mice were used as control. After 24 hours of ALI induction the cytokines were quantified by ELISA. *P < 0.05 in relation to B group; ^θ^P < 0.05 in relation to LPS group.

LPS injection increased levels of IL-1β, IL-6, IL-17A and TNF-α in the BAL, and LED treatment significantly reduced levels of IL-1β, IL-17A and TNF-α (Fig. 3A–D). Moreover, LPS injection elevated the level of IFN-γ in the BAL, and LED treatment further enhanced the concentration of this cytokine in the BAL (Fig. 3F). No alterations on IL-10 levels were observed in all groups of animals (Fig. 3E).

### The role of LED treatment in the cytokines gene expression in experimental model of ALI

**Figure 4:**
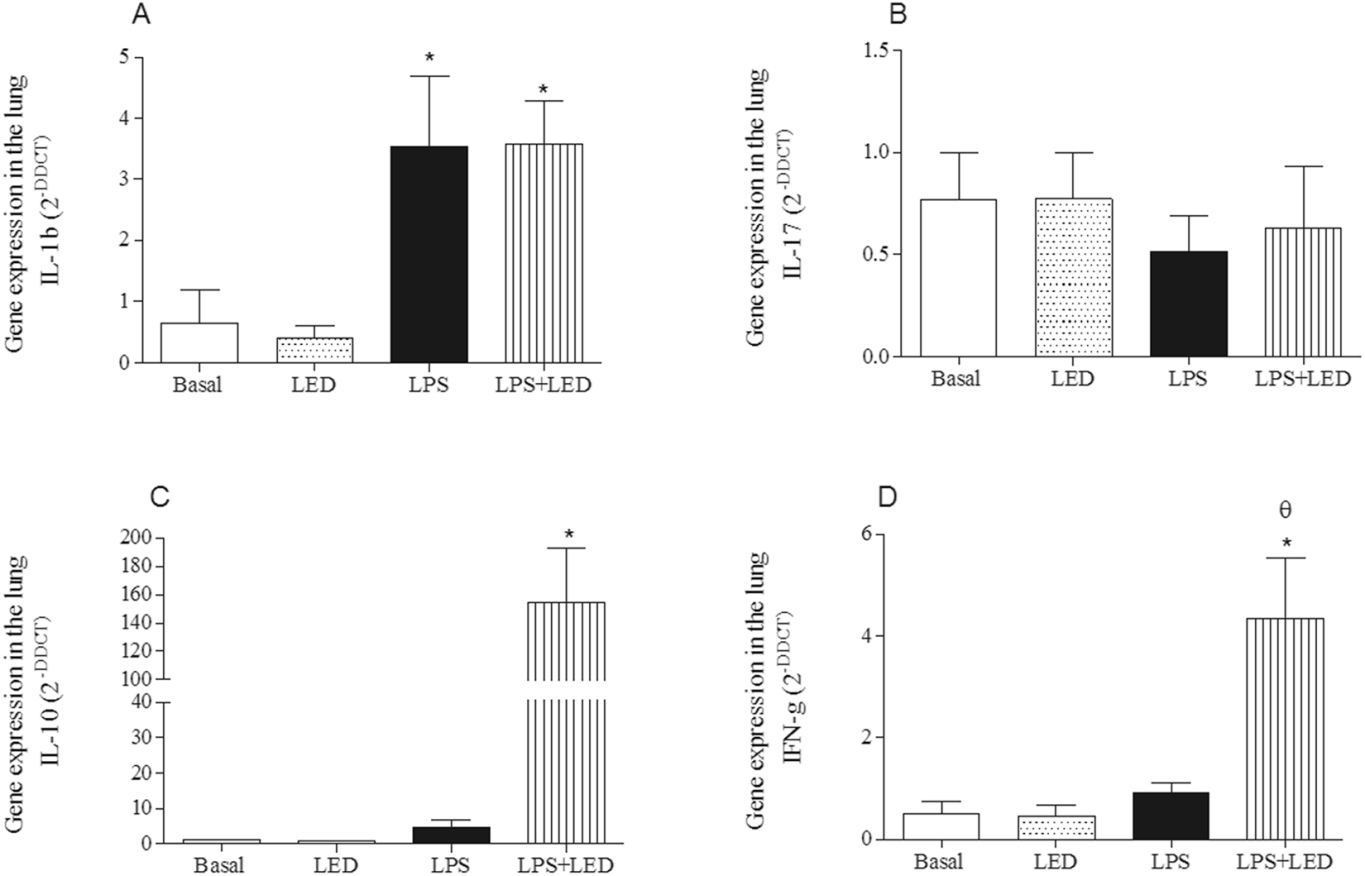
LED treatment increases the expression of anti-inflammatory cytokines without interferes in the expression of pro-inflammatory cytokines in ALI experimental model. Groups of mice were induced to ALI by IP injection of LPS and treated or not with LED 2 and 6 h after induction. Non-manipulated and LED treated mice were used as control. After 24 hours of ALI induction the gene expression was determined. Data mean ± SEM of 6 animals per group. *P < 0.05 in relation to B group; ^θ^P < 0.05 in relation to LPS group.

The analysis of gene expression on lung tissue showed that LPS injection enhanced mRNA levels of IL-1β, and did not alter the levels of IL-17, INF-γ and IL-10 (Fig. 4A–D). The treatment with LED in LPS injected animals did not reduce the elevated levels of IL-1β (Fig. 4A), and IL-17 (Fig. 4B), but markedly enhanced mRNA levels of INF-γ and IL-10 (Fig. 4C and D).

### Effects of LED treatment in the gene expression of Toll-like receptors (TLR), NF-KB translocation and annexin V expression in experimental model of ALI

**Figure 5:**
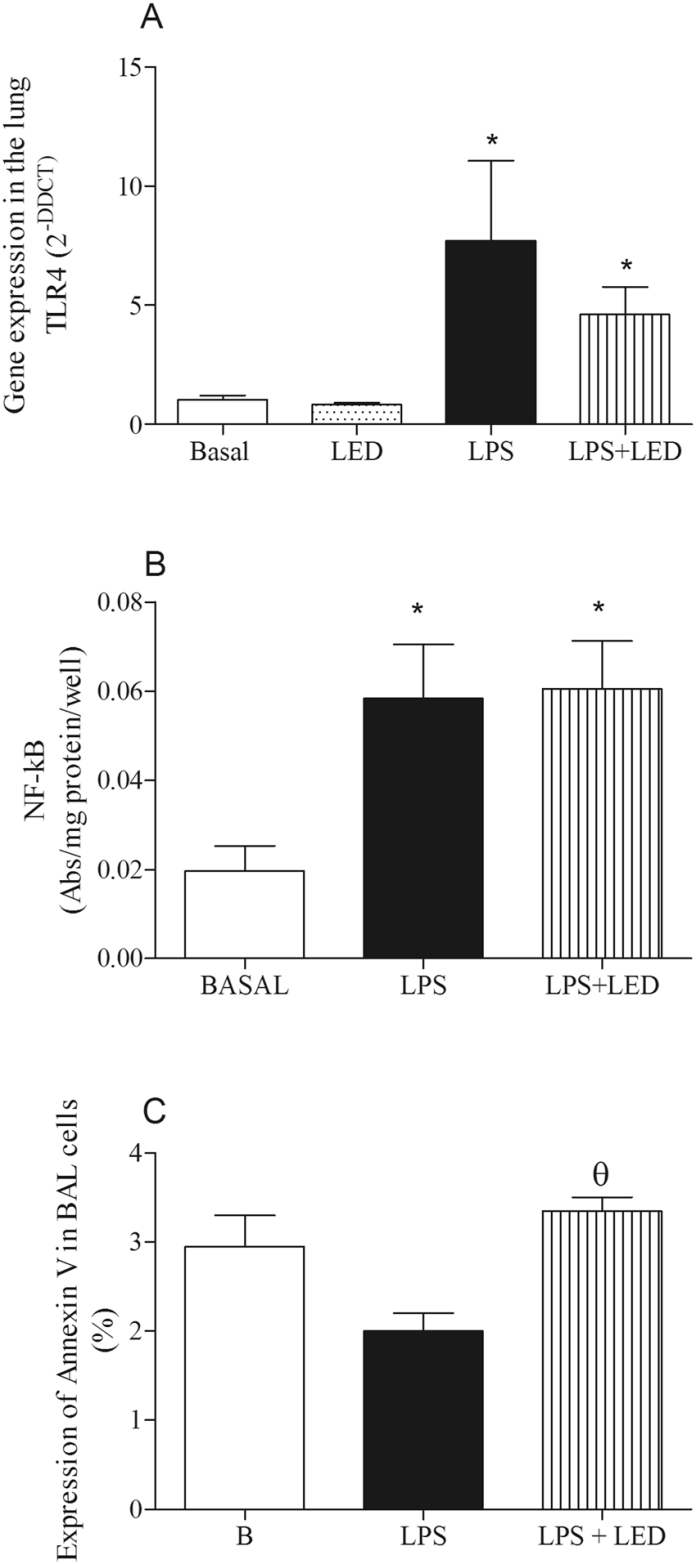
LED treatment decreases TLR4 expression without alters NF-kB translocation and elevated annexin V expression in ALI experimental model. Groups of mice were induced to ALI by ip injection of LPS and treated or not with LED 2 and 6 h after induction. Non-manipulated and LED treated mice were used as control. After 24 hours of ALI induction the TLR4 gene expression, annexin V and NF-KB translocation were determined. Data mean ± SEM of 6 animals per group. *P < 0.05 in relation to B group; ^θ^P < 0.05 in relation to LPS group.

As expected, in Fig. 5 (Panel A) gene expression of TLR4 was enhanced in LPS-treated animals and LED treatment partially reduced these levels. Values obtained were significant lesser to those in LPS-inject animals, and significant higher in comparison to those in Control animals (Basal and LED).

We also observed that LPS systemic injection enhanced the activation of NF kappa B in the lung cells, which was not reduced by LED treatment (Panel B).

In panel C we observed that LED treatment increased the expression of annexin V in relation to non-treated mice. No differences were observed between LPS and basal groups.

### Effects of LED treatment in the oxidative burst and phagocytosis in experimental model of ALI

**Figure 6:**
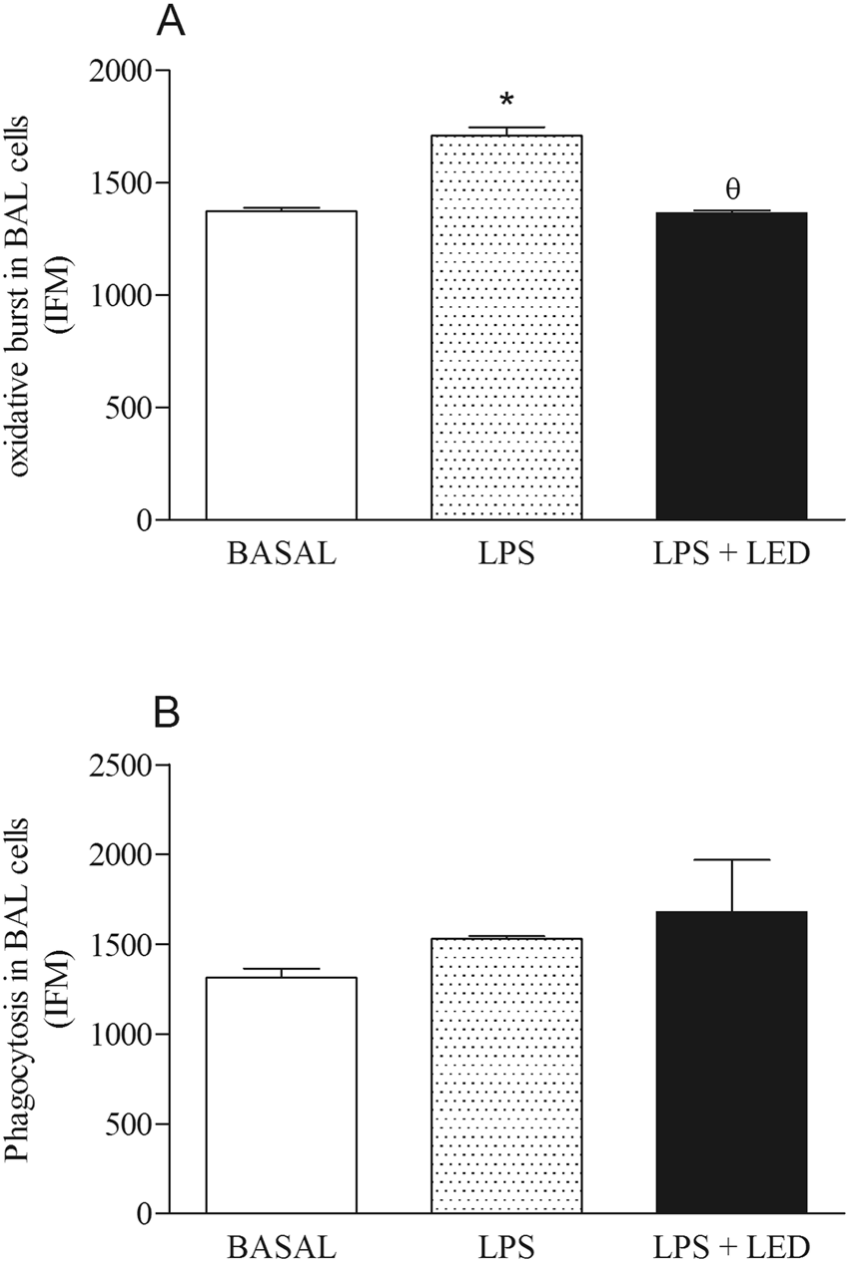
LED treatment decreases oxidative burst without alters phagocytosis in ALI experimental model. Groups of mice were induced to ALI by ip injection of LPS and treated or not with LED 2 and 6 h after induction. Non-manipulated and LED treated mice were used as control. After 24 hours of ALI induction the oxidative burst and phagocytosis were determined. Data mean ± SEM of 6 animals per group. *P < 0.05 in relation to B group; ^θ^P < 0.05 in relation to LPS group.

Data obtained in the measurement of oxidative burst confirmed the model of LPS to induce ALI, as i.p. injection of LPS enhanced the oxidative burst by BAL cells when compared to samples obtained from the Basal group. Cells collected LPS injected and LED treated animals presented reduced oxidative burst in comparison to LPS injected mice (Fig. 6A).

Furthermore, cells obtained from the BAL were tested as phagocytosis to *S. aureus*. Our data showed that phagocytes obtained from all groups of rats presented similar ability to engulf the bacteria (Fig. 6B).

### Effects of LED treatment in the peribronchiolar and perivascular infiltrated, oedema and mucus production in experimental model of ALI

**Figure 7:**
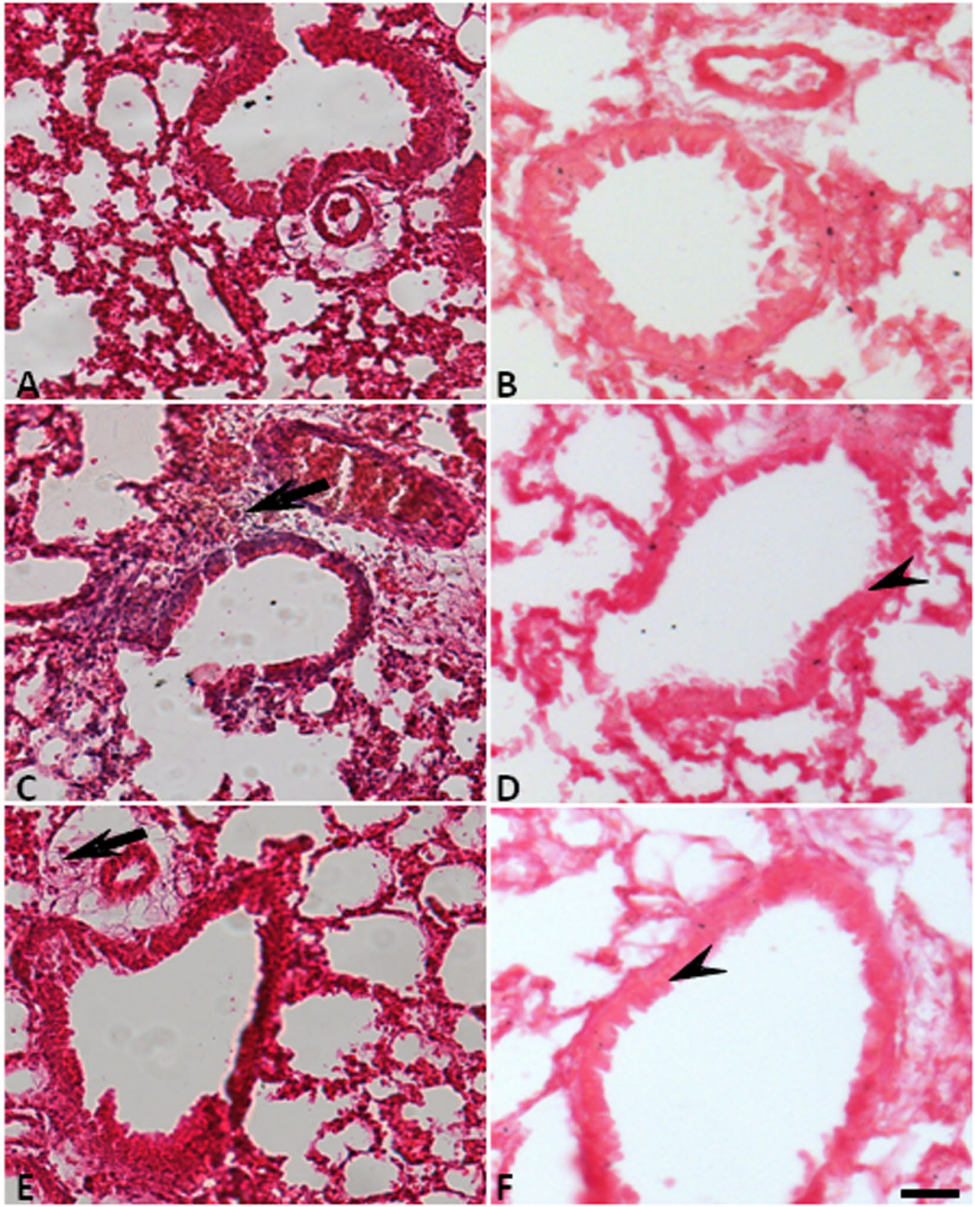
LED treatment reduces leukocytes infiltration without interfers in the mucus production in ARDS experimental model. Groups of mice were induced to ALI by ip injection of LPS and treated or not with LED 2 and 6 h after induction. Non-manipulated and LED treated mice were used as control. After 24 hours of ALI induction the histological analysis was performed. Data mean ± SEM of 6 animals per group. Coloration: (**A**,**C**,**E**) Hematoxylin and eosin, the arrows show the cellular infiltrate in the parenchyma (**B**,**D**,**F**) PAS, the arrows head show the mucus production by hyperplasia of globet cells. Bars = (**A**,**C**,**E**) 50 µm and, (**B**,**D**,**F**) 10 µm.

Figure 7 and Tables 1 and 2 show that LED treatment did not reduced the mucus production (arrow head) as wells as oedema, as values found were similar to those in LPS injected animals and much higher than those found in Control animals. Conversely, LED treatment partially reduced the perivascular and peribronchiolar cell infiltration induced by LPS injection (arrow).

**Table 1:** Parameters of LED treatment.

Wavelength	Potency	Radiant exposure	Irradiance	Area	Total energy	Exposure time
660 ± 20 nm	100 mW	5 J/cm^2^	33,3 mW/cm^2^	2,8 cm^2^	15 J	150 s

The protocol of LED irradiation was performed two and six hours after LPS injection directly in the skin on the respiratory tract (trachea and lungs) during 150 s.

**Table 2:** LED treatment did not reduce the mucus production as wells as oedema, but partially reduced the perivascular and peribronchiolar cell infiltration after LPS injection.

Groups	Oedema	Mucus	Peribronchiolar infiltrate	Perivascular infiltrate
Basal	0.17 ± 0.02	0.33 ± 0,03	0.17 ± 0.02	0.17 ± 0.02
LED	0.33 ± 0.03	0.33 ± 0.3	0.33 ± 0.03	0.33 ± 0.03
LPS	2,60 ± 0,3*	2,40 ± 0,2*	1.20 ± 0.1*	2.40 ± 0.3*
LPS+LED	2,20 ± 0,2*	2,20 ± 0,2*	0.8 ± 0.1*^θ^	1.80 ± 0.2*^θ^

Groups of mice were induced to ALI by ip injection of LPS and treated or not with LED 2 and 6 h after induction. Non-manipulated and LED treated mice were used as control. After 24 hours of ALI induction the histological analysis was performed. Data mean ± SEM of 6 animals per group. *P < 0.05 in relation to B group; ^θ^P < 0.05 in relation to LPS group.

## Discussion

Using an experimental model of sepsis, we have shown, for the first time, that short periods of local LED treatment was effective in treating ALI, by impairing the neutrophil influx, and consequently, reducing inflammation in the lung.

Photobiomodulation has shown promissory effects in several lung diseases, including ALI^15–22^. By using lung inflammatory models, we have shown that treatments with low level laser therapy (LLLT) have reduced the development of neutrophilic lung inflammation that was induced by formaldehyde (FA), as observed by the reduced number of leukocytes, mast cell degranulation, and MPO activities, in the lung^16,17^. Moreover, LLLT also reduced the microvascular lung permeability in the parenchyma and in the intrapulmonary bronchi. Furthermore, it reduced and increased the levels of inflammatory and anti-inflammatory cytokines, respectively, in the BAL, and it favored the expression of antioxidant enzymes in the lungs^17^. Together, our results have confirmed the efficacy of LLLT on inflammation, by affecting several parameters of lung inflammation that were evoked by a pollutant.

LED (light emitting diode) is semiconductor diode that has been used to treat several pathologies. LED emission of light is monochromatic, non-coherent and it is not collimated. It differs from LLLT^28^. Despite these differences, treatments with LED may be considered as being as efficient as those treatments with laser therapies, since the coherence of Laser light seems to not be responsible for the therapeutic effects^29^. Therefore, this investigation into the therapeutically properties of LED has now been pointed out as being an effective alternative to laser, while exerting similar effects and with lower cost^30^.

The investigators chose to study LED radiation in the visible (660 nm) regions of the red color spectrum, because in earlier studies, this spectrum has shown beneficial effects for the treatment of lung disease^21,22^. Be that as it may, this same visible region for laser has also demonstrated excellent effects^15,16,19,20^. We used the visible red color in our studies because several studies of the literature have shown that the wavelength of visible red is more effective to treat tissues and organs, which are deeply localized, as lungs. Other wavelengths such as blue, green are not efficient to treat these organs because the lights are not capable to penetrate in the tissue. In addition, recent study showed that blue light induced oxidative stress in live skin^31^. In order to confirm that the red color, in fact, is the best wavelength to treat lung disease, we treated the animals with green LED that wavelength is 520 nm. Results obtained showed that green LED did not reverse the increased cell influx into the alveolar space as well as inflammatory cytokines such as IL-17, IL-1beta and TNF-alpha (supplementary data). This evaluation showed clearly that the effects of LED used in our studies are dependent on the wavelength and confirmed that the resonance of light in the lung is essential to the beneficial effects of LED.

To the best of our knowledge, the role of LED on ALI has not been shown. We have investigated the role of short period of LED treatment at the onset of ALI. In this model of sepsis, neutrophils were the main cells that were recruited into the inflammatory exudates and lung tissues during the first 24 hours. Macrophages are resident cells in the lung and lymphocytes represent a small population that is recruited into the lung and BAL in the early phase of sepsis. Consequently, **o**ur data has clearly shown that short period LED treatment during the progression of early phase of ALI inhibited the influxes of leukocytes into the lung. The impaired leukocyte amounts in the BAL reflected the reduced infiltration of neutrophils, as we also found reduced activities of MPO. Moreover, it is known that neutrophils are recruited into the lung during the first 24 hours of ALI^8^. Therefore, the actions of LED on neutrophils recruitment into the lung may be pivotal, in order to reduce tissue damage, when considering the relevance of these cells in ALI^8^. Indeed, the amount of pro-inflammatory cytokines in the BAL was reduced, which may be due to a lower amount of leukocytes in the lungs, especially neutrophils, in the early phase of ALI, or it may due to an impaired activation of the lung cells by the products that are released by the neutrophils.

What is more, we have also shown that although LED treatment occurs locally in the lungs and the trachea, it also altered the profile of neutrophils in the blood. It is well known that sepsis causes leukopenia, which is characterized by a decline of the mononuclear cells, but it enhances the neutrophils in the blood that are mobilized from the peripheral and medullar pools^32^. Indeed, LPS administration has led to blood alterations and LED treatment has reversed the neutrophilia. The lung microcirculatory pool is the mainly responsible for the rapid growth of neutrophilia after an infeccion^33,34^. One may suppose that local application of LED may have impaired a detachment of the neutrophils from the pulmonary post capillary vessels and their migration into the circulating blood. These original hypotheses were reinforced by the inability of the LED treatment to reverse the reduced number of mononuclear cells in the blood, as well as the leukopenia in the bone marrow.

As previously has been mentioned, the levels of the pro-inflammatory cytokines in the BAL were reduced after the LED treatment. However, the IFN-γ levels were enhanced by the LED treatment in the BAL of the LPS-injected mice. IFN-γ is an important Th1 associated pro-inflammatory cytokine that inhibits the leukocyte influx into the lungs and it is mainly secreted by lymphocytes^35,36^. Moreover, IFN-γ secretion secretion is also important during a resolution of inflammation and it is responsible for a limitation of inflammation in a tissue injury^37^.

Our data has further supported that anti-inflammatory cytokines take part in the anti-inflammatory actions of a LED treatment, as was detected by higher levels of mRNA IL-10 and IFN-γ in the lung of the mice. IL-10 is secreted by the lymphocytes, the monocytes, and macrophages, when halting the inflammatory process. It has been shown that administrations of recombinant IL-10 confer significant therapeutical protection in experimental models of sepsis. These effects may be a consequence of the pronounced anti-inflammatory repercussions of IL-10 on the neutrophils. IL-10 inhibits IL-1β, TNF-α, and IL-8 production. It also blocks the cytokine-induced chemotaxis and the oxidative burst, and therefore, it interferes with the neutrophil-mediated tissue injuries^38,39^.

LPS interacts with TLR4, which is constitutive in the cell membrane. It is up-expressed under activation^5^ and downstream there is a cascade of intracellular transcriptions of the inflammatory genes. In this study, we have shown that LPS injections elevated the TLR-4 expression and the NFκB translocation into the nuclei, clearly indicating that LPS binding to the TLR-4 induces MyD-88 activation, Interleukin 1 Receptor Associated Kinase (IRAK-1 and 4), TNF receptor-associated factor 1 (TRAF), with a consequent translocation of the NF-κB into the nuclei for the gene transcription. This sequence of events is fundamental for the infectious inflammatory responses and our data showed that the TLR4 expression and the translocation of NF-κB into the nuclei were not down-regulated by the LED treatment. This data may show that LED treatment does not affect the activation of the cells in the lungs by the LPS treatment and the data corroborates that the primordial action of acute local LED treatment are an inhibition on the neutrophil influxes.

Following this line of evidence, we have investigated the ability of LED treatment to modify the oxidative burst and the phagocytosis of phagocytes that were present in the BAL. LED treatment impaired the ability of the lung cells to generate ROS, but they did not affect the phagocytic activity of the phagocytes. Together, this data has shown that the LED treatment did not impair the ability of the phagocytes to engulf bacteria and to contribute to the resolution of sepsis. However, they reduced the oxidative stress burst, which may have reduced the release of the amount of ROS into the tissues. It is known that a high ROS is produced in the early phase of ALI, mainly by migrated neutrophils, and that this leads to tissue damage, cell dysfunction and uncontrolled inflammatory responses^40,41^. Thus, by reducing the production of ROS, this impaired a diversity of the signaling pathways on inflammatory cells and these mechanisms may contribute to the reduced inflammatory response.

Mucus and oedema are hallmarks of ALI and they are a correlation of the inflammatory cell activations. Hence, we expected that both of the parameters of ALI would be reduced by the LED treatment. The mucins are synthetized and are secreted by the epithelial goblet cells into the submucosal glands of the airways^42^. An increased release of mucus is mainly mediated by IL-14 and IL-13 when secreted by the resident and migrated mast cells^42^. The oedema is caused by enhanced vascular permeability, as a result of the contractions of the endothelial cells from the post capillary venules that are mediated by a diversity of chemical mediators, such as amines, active complement system fractions, cytokines and prostanoids^43,44^. Therefore, LED treatment did not interfer with these mechanisms and this data further supported the role of LED treatment on neutrophil in the lungs.

When associated, our data has clear shown that short-duration, local treatment with LED impaired the ALI development that was induced by sepsis. The main mechanisms of action were the interference on the cell mobilization of the cells into the lungs, especially the neutrophils, which exacerbated the influxes and the inflammatory activities that were damaging the lung tissue. As a consequence, we are confident to propose that local LED treatment be a co-adjuvant, non-invasive and inexpensive approach for sepsis treatment.

## Material and Methods

### Animals

Male Balb/c mice were maintained in light and temperature-controlled room (12/12-hour light-dark cycle, 21 ± 2 °C), with free access to food and water. Groups of mice were killed by sectioning the abdominal aorta under deep anaesthesia with ketamine-xylazine by intraperitoneal route (100 mg/kg and 20 mg/kg, respectively) 24 hours after the LPS or saline injection.

#### Ethical Approval and accordance

The animals were obtained from the University Nove de Julho and the experiments were approved by the Animal Care Committee University Nove de Julho (CoEP-UNINOVE, AN0006/2015). The methods used in the present study were carried out in accordance with the relevant guidelines and regulations.

### Induction of experimental model of acute lung inflammation (ALI)

The animals were submitted to injection of lipopolysaccharide (LPS, *Samonella abortus equi*, 5 mg/Kg, ip) or vehicle (saline) to induction of ALI. The dose chosen was based on the sub-septic effects of LPS^27^.

### Protocol of Light-Emitting Diode (LED) treatment

The protocol of LED irradiation was performed two and six hours after LPS injection directly in the skin on the respiratory tract (trachea and lungs) during 150 s according earlier studies^21^. Thus, there were not distance between the animal and the spot of LED. The wavelength used in this study includes the range visible of electromagnetic corresponding to the red color. The parameters of LED are following above:

### Groups of study

The animals were randomized in 4 groups:

Basal group: Non-manipulated animals.

LPS group: Animals treated with LPS injection.

LED group: Animals treated with saline injection and irradiated with LED.

LPS+LED group: Animals treated with LPS injection and irradiated with LED.

### Evaluation of inflammatory cells migrated to the bronchoalveolar lavage (BAL) and lungs

The total number of inflammatory cells migrated to BAL were determined according to earlier studies^15,21,27^. Moreover, histological analyses were also performed in order to determine the peribronchiolar and perivascular infiltration. Lung tissues were stained with hematoxylin and eosin for morphological analysis.

We also quantified the myeloperoxidase (MPO) activity in the lung tissue. This evaluation was used as an index of the presence of local neutrophils, according to previous studies^26,27^.

### Evaluation of oedema and mucus production in the lung

Histological analyses were performed in order to determine the edema and mucus production in the lungs. Lung tissues were stained with hematoxylin/eosin and PAS (PerodicacidShiff) for edema and mucus analysis respectively. In addition, oedema was also evaluated by Wet/dry weight ratio.

### Determination of cytokines released in the BAL fluid as well as the gene expression in the lung

Cytokines levels were determined according to the manufacturer’s specifications using ELISA kits purchased from Biolegend (San Diego, USA) in the BAL supernatant samples. Results were expressed as pg of cytokine produced per ml. The assays were made in duplicate for every sample using standard curves for IL-1β, IL-6, TNF-α, IL-17, IL-10, and IFN-γ.

Gene expression of cytokines in the lung tissue was determined according to Silva Macedo *et al*. (2016). RT-PCR was performed using Taqman real-time PCR assay (Applied Biosystem, USA) for the following molecules: IL-6 (Mm00446190_m1), IL-10 (Mm01288386_m1), TNF-α: (Mm00443258_m1), IL-1β (Mm00434228_m1), IL-17(Mm01189488_m1), IFN-gamma (Mm01168134_m1), and HPRT (Mm00446968_m1). Sequence Detection Software 1.9 (SDS) was used for the analysis and mRNA expression was normalized to HPRT expression.

### Quantification of phagocytosis and oxidative burst in BAL cells

We used BAL cells (4 × 10^4^ cells/well) for the analysis of phagocytosis and oxidative burst. Direct measurements of the fluorescence mean estimated by means of propidium iodide (PI)-labeled *S. aureus* and 2′,7′-dichlorofluorescin diacetate (DCFH-DA) fluorescence cells were recorded as phagocytosis and oxidative burst respectively.

A flow cytometer (FACS Calibur, Becton Dickinson Immunocytometry Systems, San Jose, CA, USA) interfaced with a Macintosh G4computer was used. Data from 10,000 events were collected in list mode and analyzed in Cell Quest (Becton Dickinson Immunocytometry Systems). Fluorescence data were plotted on log scale. Green fluorescence from DCFH was measured at 530 ± 30 nm (FL1 detector) and red fluorescence from PI-*S. aureus* was measured at 585 ± 42 nm (FL2 detector).

### Evaluation of translocation of the transcription factor NF-κB and TLR4 gene expression

To evaluate the translocation of the transcription factor NF-κB from the cytoplasm to the nucleus, nuclear extracts from the lungs were prepared in accordance with the manufacturer’s instructions (Cayman Chemical, Ann Arbor, MI). The gene expression of TLR4 was performed as describe above.

### Evaluation of apoptosis

The total number of BAL cells was quantified using a Neubauer chamber. After that, BAL cells (1 × 10^4^ cells) were incubated with Annexin V conjugated to FITC (1:100) for 20 min to evaluate cell apoptosis. The BAL cells population was characterized by different size and complexity parameters of different cell types detected by BD Accuri^TM^ C6 flow cytometry (Becton, Dickinson and Company, USA). The optical signals emitted were converted into electronic signals and were analyzed by FlowJo software (Tree Star, Inc., Ashland, TN, USA). Results of apoptosis were shown as the percentage of Annexin V positive cells.

## Electronic supplementary material

Supplementary information
