# Supplementary material for: Beneficial effects of Red Light-Emitting Diode treatment in experimental model of acute lung injury induced by sepsis
Source: Sci Rep. 2017 Oct 4;7:12670. doi: 10.1038/s41598-017-13117-5 (PMC5627274; doi:10.1038/s41598-017-13117-5)
Supplement: Supplementary file 1 — Supplementary information [file 41598_2017_13117_MOESM1_ESM.pdf]

Supplementary information

Beneficial effects of Red Light-Emitting Diode treatment in experimental model of acute lung injury induced by sepsis

Silvia Goes Costa[1], Éric Diego Barioni[2], Aline Ignácio[3], Juliana Albuquerque[1], Niels Olsen Saraiva Câmara[3], Christiane Pavani[1], Luana Beatriz Vitoretti[1], Amílcar Sabino Damazo[4], Sandra Helena Poliselli Farsky[2], Adriana Lino-dos-Santos-Franco[1*].

Material and methods:

Evaluation of migrated cells in the BAL and MPO activity

The total number of inflammatory cells migrated to BAL were determined according to earlier studies[15,21,27]. We also quantified the myeloperoxidase (MPO) activity in the lung tissue. This evaluation was used as an index of the presence of local neutrophils, according to previous studies[26,27].

Determination of IL-17, IL-1β and TNF- α in the BAL fluid

Cytokines levels were determined according to the manufacturer's specifications using ELISA kits purchased from Biolegend (San Diego, USA) in the BAL supernatant samples. Results were expressed as pg of cytokine produced per ml. The assays were made in duplicate for every sample using standard curves for IL-1β, TNF-α and IL-17.

Results:

Data presented in Table S1 show that the treatment with green LED in LPS injected mice did not reverse the increased number of leukocytes recruited into the BAL while red LED was capable to reduce this augment. Similarly, the elevated activity of MPO was not reduced by treatment with green LED (Table S2).

In table S3 we can observe that green LED treatment in LPS injected mice did not reduced the levels of IL-17 and IL-1beta in the BAL fluid, while TNF-alpha levels were increased.

Number of cells recruited in the BAL (bronchoalveolar lavage) after treatment with green LED:

**Groups (n=5)**	**Total number of cells (x10^4 cells/ml)**
**LPS**	17,65
**Red LED**	5,31*
**Green LED**	22,5

Table S1. Evaluation of green LED in the cells recruited in the BAL Groups of mice were induced to ALI by ip injection of LPS and treated with red LED or green LED 2 and 6 h after the induction. LPS mice were used as control. After 24 hours of ALI induction, the cellular recruitment was determined. Data mean ± SEM of 5 animals per group. *P<0.05 in relation to LPS group.

Myeloperoxidase (MPO) activity in the lung tissue after treatment with green LED:

**Groups (n=5)**	**MPO activity (absorbance)**
**LPS**	6,75
**Red LED**	3,3*
**Green LED**	6,98

Table S2. Evaluation of green LED in the MPO activity in the lung tissue. Groups of mice were induced to ALI by ip injection of LPS and treated with red LED or green LED 2 and 6 h after the induction. LPS mice were used as control. After 24 hours of ALI induction, the MPO activity was determined. Data mean ± SEM of 5 animals per group. *P<0.05 in relation to LPS group.

Inflammatory cytokines in the BAL after treatment with green LED:

Groups (n=5)	IL-17 (pg/ml)	IL-1beta (pg/ml)	TNF-alpha (pg/ml)
**LPS**	302	1015	322
**Red LED**	194*	298*	74*
**Green LED**	273	1300	800*

Table S3. Evaluation of green LED in the cytokines released in the BAL fluid. Groups of mice were induced to ALI by ip injection of LPS and treated with red LED or green LED 2 and 6 h after the induction. LPS mice were used as control. After 24 hours of ALI induction, the cytokines were quantified. Data mean ± SEM of 5 animals per group. *P<0.05 in relation to LPS group.
